# Inline gamma spectroscopy and liquid scintillation HPLC reveal daughters in ^225^Ac-radiopharmaceutical

**DOI:** 10.1186/s41181-026-00476-6

**Published:** 2026-07-21

**Authors:** Guilhem Claude, Matthias Balzer, Winfried Brenner, Frank Bruchertseifer, Alfred Morgenstern, David Thonon, Sarah Spreckelmeyer

**Affiliations:** 1 Klinik Für Nuklearmedizin, Radiopharmazie, Charité—Universitätsmedizin Berlin, corporate member of Freie Universität Berlin and Humboldt-Universität Zu Berlin, Augustenburger Platz 1, 13353 Berlin, Germany; 2 European Commission, Joint Research Centre, Karlsruhe, Germany; 3 Elysia-Raytest, Rue du Bois Saint-Jean 23, 4102 Ougrée, Belgium

**Keywords:** HPLC, Target alpha therapy, Theranostics, Ac-225

## Abstract

**Background:**

Analytical data for ^225^Ac-labeled radiopharmaceuticals are currently obtained using thin-layer chromatography and high-performance liquid chromatography, the latter followed by offline fraction collection and delayed gamma-counting after secular equilibrium with gamma-emitting daughters has been reached. These procedures are slow and do not depict the distribution of daughter radionuclides. An inline HPLC detection setup combining liquid scintillation detection and LaBr_3_-based gamma-spectroscopy is presented to enable faster and more informative analysis of a ^225^Ac radiopharmaceutical.

**Results:**

Four chromatographic signals were identified corresponding to uncomplexed ^221^Fr with at most a minor contribution of free ^213^Bi, [^213^Bi]Bi-PSMA I&T, [^225^Ac]Ac-PSMA I&T, and [^209^Pb]Pb-PSMA I&T. The retention times corresponded to those of the non-radioactive analogs determined by UV detection. Assignments were confirmed by fraction collection followed by gamma-spectroscopy and beta liquid scintillation counting. The distribution of these species changed upon heating the reaction mixture or after addition of another ligand.

**Conclusions:**

The combined detection setup enables rapid observation of isotope distribution and daughter behavior during chromatographic analysis and avoids fraction handling by operating as a closed system, thereby reducing contamination risk and radiation exposure. This approach may contribute to improved quality control procedures for ^225^Ac radiopharmaceuticals and other alpha-emitting radionuclides. At the present stage, however, it should be regarded as a qualitative to semi-quantitative proof-of-principle.

**Supplementary Information:**

The online version contains supplementary material available at 10.1186/s41181-026-00476-6.

## Introduction

^225^Ac-labeled ligands e.g. with prostate-specific membrane antigen (PSMA) for treatment of prostate cancer and DOTA-TATE/TOC for neuroendocrine cancers are gaining clinical importance. The high linear energy transfer of alpha-particles may lead to higher cytotoxicity at the micrometer range while preserving distant tissues. This leads to a marked response in patients, including those previously treated with the corresponding ^177^Lu-analogs (Rosar et al. 2021; Shi et al. 2022; Chan et al. 2017). At the moment, a growing number of clinical trials involving ^225^Ac-labeled radiopharmaceuticals have been registered. However, the decay chains of alpha-emitters with properties suitable for therapeutic use are considerably more complex than that of lutetium (Toro-Gonzalez et al. 2026; Tronchin et al. 2024).

**Fig. 1:**
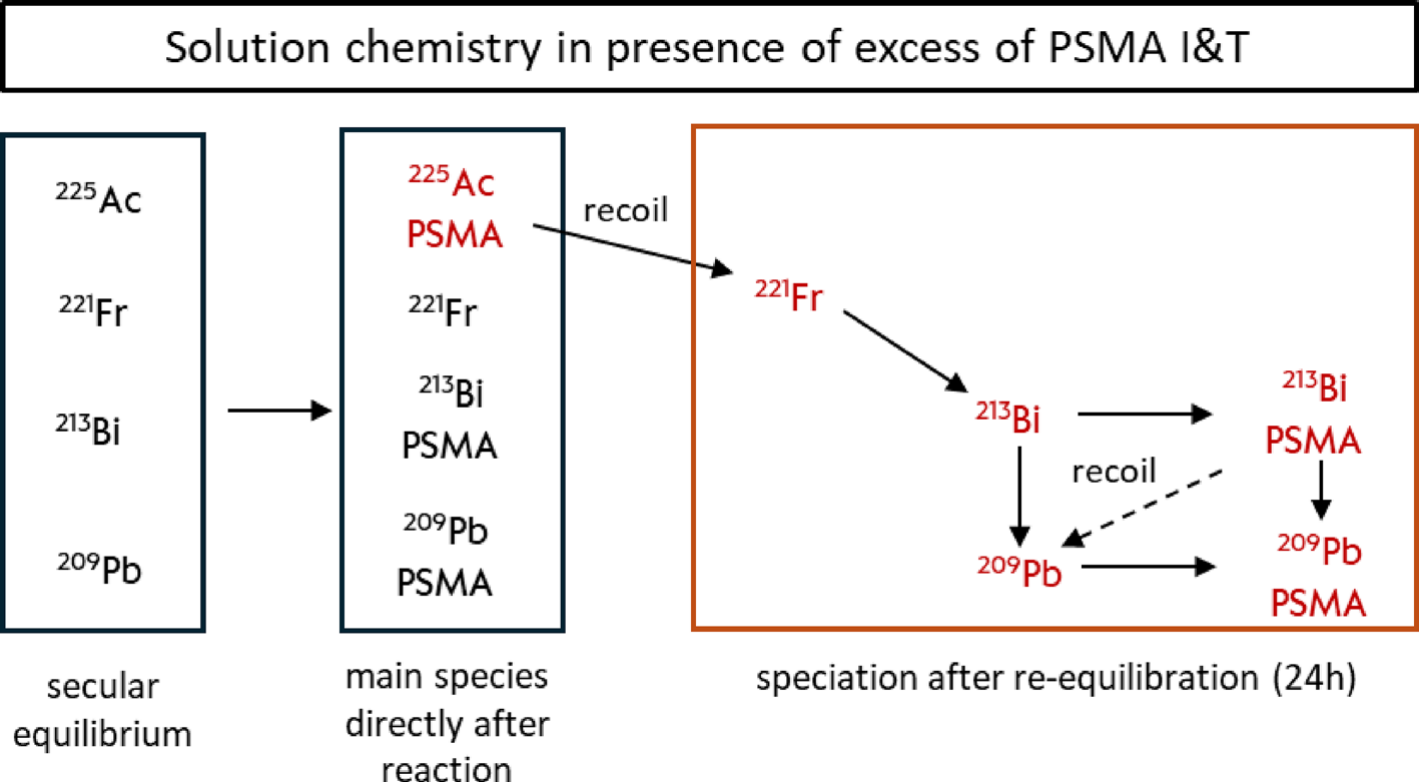
Proposed product formation of PSMA I&T complexes with a ^225^Ac-solution in secular equilibrium with an excess of ligand. For clarity only analytically relevant isotopes are depicted

^225^Ac decay comprises seven radionuclides emitting alpha, beta, and gamma radiation with half-lives ranging from milliseconds to hours. With the present inline detection setup, only ^221^Fr (t_1/2_ = 4.8 min), ^213^Bi (t_1/2_ = 45.6 min), ^225^Ac (t_1/2_ = 9.92 d), and ^209^Pb (t_1/2_ = 3.25 h) can be detected as separate species (see Fig. 1). The short-lived daughters ^217^At (t_1/2_ = 32 ms) and ^213^Po (t_1/2_ = 4 µs), originating from ^221^Fr and ^213^Bi, respectively, decay completely within the residence time in front of the detector due to the cell size and the HPLC flowrate and therefore cannot be distinguished from their parent isotopes. ^209^Tl (t_1/2_ = 2.2 min) is generated in only about 2% of ^213^Bi decays and consequently contributes negligibly to the recorded signal (Suliman et al. 2013). Upon decay, daughter nuclides can be released from the chelate by both the recoil effect and changes in the coordination properties of the daughter nuclide compared to its parent (e.g. Ac being an actinide with a charge of + 3 while Fr is an alkali metal with a charge of + 1) (Asano et al. 1974; Kruijff et al. 2019; Zitzmann-Kolbe et al. 2025; Asano and Taniguchi 1978; Mirzadeh et al. 1993). The re-chelation of recoiled daughters with ligands is not limited by the stoichiometry of the reactions as ligands are nearly always present in significant excess in the reaction solution. The re-chelation of daughters will more likely depend on other parameters as it has been shown by Larenkov et al. that complexation reactions of e.g. ^177^Lu with DOTA can happen at room temperature, depending on the buffer and its concentration (Maguire et al. 2014; Larenkov et al. 2024).

The analysis of a complex decay chain with several alpha and beta emitters with weak gamma components and a dynamic chemistry in solution presents a significant analytical challenge. Radio-thin layer chromatography (TLC) has been proposed as an analytical method for ^225^Ac radiopharmaceuticals and can be read directly on standard NaI radio-TLC scanners, while alpha- and beta-sensitive detection approaches have also been reported (Yang et al. 2020; Kelly et al. 2021; Kleynhans and Duatti 2022; Castillo Seoane et al. 2022; Pretze et al. 2024). TLC does provide a fast and reliable distinction between any complexed and “free” nuclides (e.g., “free”/buffer-complexed metal or colloids) (Pretze et al. 2021). However, a TLC cannot distinguish other side products such as radiolytically induced modifications and cannot provide a clear speciation to confirm the presence in solution of the correct target radiopharmaceutical (Hooijman et al. 2025; Mu et al. 2013; Schmitl et al. 2023).

**Fig. 2:**
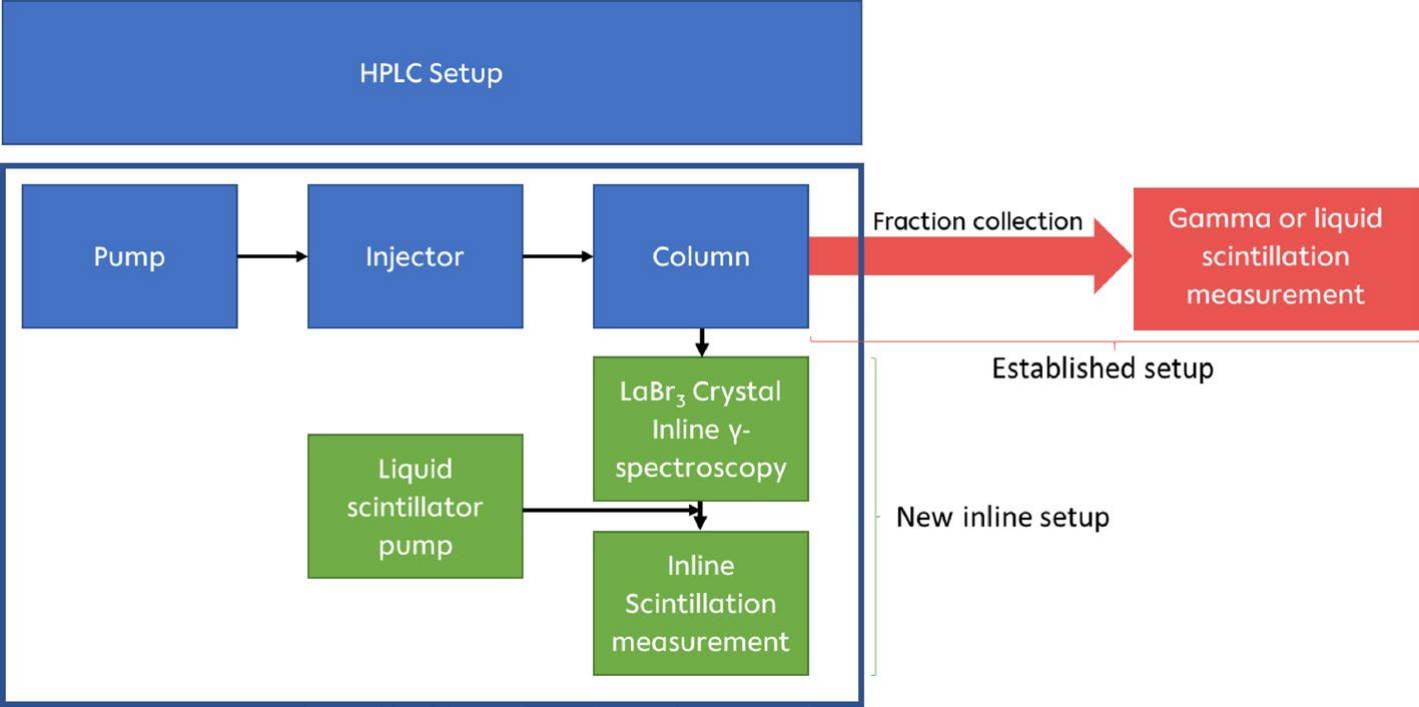
Flow chart comparing our inline setup with fraction collections

High performance liquid chromatography should, in principle, provide the required greater chemical specificity, but with the same limitation of standard gamma detectors it likewise depends on daughter emission; consequently, the published procedures require collection of fractions and measurement by gamma-spectroscopy after reaching secular equilibrium (Abou et al. 2022; Hooijman et al. 2021, 2024). This workaround is slow, compromises chromatographic resolution, heightens contamination and radioprotection risks, delays product release by several hours, introduces potential for fraction mix-ups, and remains incapable of capturing extremely short-lived or pure alpha/beta intermediates (e.g., ^209^Pb) in real time. Hence, an integrated inline detection system covering both gamma and beta/alpha emissions is desirable to achieve continuous, real-time radionuclide speciation without interrupting the chromatographic process for fraction collection (see Fig. 2) (Meinard et al. 1985; Mackey et al. 1981). The hypothesis of this study is that combining inline gamma-spectroscopy and liquid scintillation detection with HPLC can provide more direct information on parent and daughter speciation during chromatographic analysis than fraction collection followed by delayed gamma-counting.

In this work, we integrate liquid-scintillation counting (LSC) and LaBr_3_-based gamma-spectroscopy as analytical techniques in our HPLC system to enable the acquisition of valuable real-time data. LSC is a well-established and effective method for measurement of both beta and alpha emitters across a wide energy range (Rapkin 1993; Hou 2018). Its high efficiency for beta and alpha radiation makes it suitable for isotopes such as ^213^Bi and ^209^Pb. LaBr_3_ gamma-spectroscopy has been shown to have a much higher spectral resolution than NaI detectors, without requiring cooling as a semi-conductor detector would and with a high response rate (Garnett et al. 2017). Thus, coupling LSC with energy-resolved LaBr_3_ gamma-spectroscopy yields complementary information: the scintillation cell measures beta/alpha activity, whereas the gamma-detector identifies photon energies specific to each daughter, provided the daughter emits gamma radiation.

## Methods

### General information

^225^Ac was obtained from the Joint Research Centre Karlsruhe as a dried chloride salt and had been separated from ^229^Th at least one day before the complexation reaction. GMP grade PSMA I&T and non-radioactive Bi-PSMA-I&T, La-PSMA-I&T and Pb-PSMA-I&T were obtained from piChem (Grambach, Austria). Tris, hydrochloric acid, sodium ascorbate and DTPA were purchased from Merck (Germany) in the highest available purity grade and were used without further purification. The ^209^Pb-containing fraction was measured using a Revvity Tri-Carb 4910 TR and ^221^Fr, ^213^Bi and ^225^Ac were measured using a Canberra Osprey-DTB.

### HPLC conditions

The HPLC measurement was performed using an Agilent 1260 Quaternary pump with 1260 VWD UV detector set at 200 nm equipped with a 14 µL UV cell (Agilent Technologies, Santa Clara, USA). An Agilent Poroshell C18 2.7 µm 100 × 4.6 mm 120 Å column was used. The following previously reported and subsequently adapted gradient was used: Solvent A acetonitrile, solvent B phosphate buffer dihydrate (3.12 g/L) adjusted to pH 2.5 with phosphoric acid with a flow set at 1.2 mL/min: 0–1.5 min 20% solvent A, 1.5–6.5 min 20–25% solvent A, 6.5–7.5 min 25–60% solvent A, 7.5–9.5 min 60% solvent A (Kraihammer et al. 2023). The temperature was not controlled. 20 µL with 200–300 kBq were injected using a manual injector per measurement. The UV trace of a synthesis is available in the supporting information (see Fig. S1) as well as the LSC trace of a replicate run (see Fig. S2).

An Elysia-Raytest Gabi Nova (Straubenhardt, Germany) detector was used either in a “conventional” total count mode over a defined energy range or using the “3D” mode to measure gamma-spectrum over 2000 keV range in a ten second repetition interval (spectrum acquisition frequency during HPLC runtime is a method parameter). A Ramona LS-Pump was used to pump Gold Star liquid scintillator (Hidex, Turku, Finland) at a rate of 3.6 mL/min, corresponding to a 1:3 ratio of HPLC eluate to scintillation cocktail, which was mixed using a T-junction with the eluate from the column. The mixture was subsequently measured in an Elysia-Raytest Ramona Star using a specific liquid scintillator cell with a volume of 200 µL. Gold Star was selected because it is our standard scintillation cocktail for mixed alpha/beta measurement and is compatible with the presence of salts originating from the HPLC buffer system. Two lower cocktail flow rates were also tested and gave acceptable results with comparable sensitivity, but a systematic optimization study was not carried out as only a limited amount of activity was available. The liquid scintillation detector signal was recorded continuously with an acquisition frequency of 1 Hz. The signals were analyzed using the software Gina X. The UV detector is integrated in the HPLC stack, whereas the radiodetectors and the scintillator pump are positioned downstream of the UV-detector and connected through a long capillary. This introduces a small but reproducible delay of the radioactive signals relative to the UV trace, which accounts for the retention-time offset between the UV and radioactive peaks.

### [^225^Ac]Ac-PSMA I&T synthesis

[^225^Ac]Ac-PSMA I&T was synthesized according to a published procedure (Hooijman et al. 2021). Briefly, a solid ^225^Ac sample (40–60 MBq) was dissolved within 30 min by using 30 µL of 0.1 M HCl at room temperature. An aliquot of 5–10 µL of that solution was added to a mixture of Tris Buffer (0.1 M, pH 9, 300 µL) and sodium ascorbate (1 M, pH 5.8, 200 µL) and PSMA I&T dissolved in water (1 mg/mL, 0.7 mM, 100 µL). The resulting mixture was then heated at 95 °C for 5 min using a Biotage Initiator microwave. An aliquot was taken to perform HPLC measurements which were performed from 15 min to 24 h (see SI) after end-of-synthesis. Then DTPA (1.5 g/L, 3.8 mM, 40 µL) and sodium ascorbate (1 M, pH 5.8, 500 µL) were added to obtain the final formulation.

## Results

**Fig. 3:**
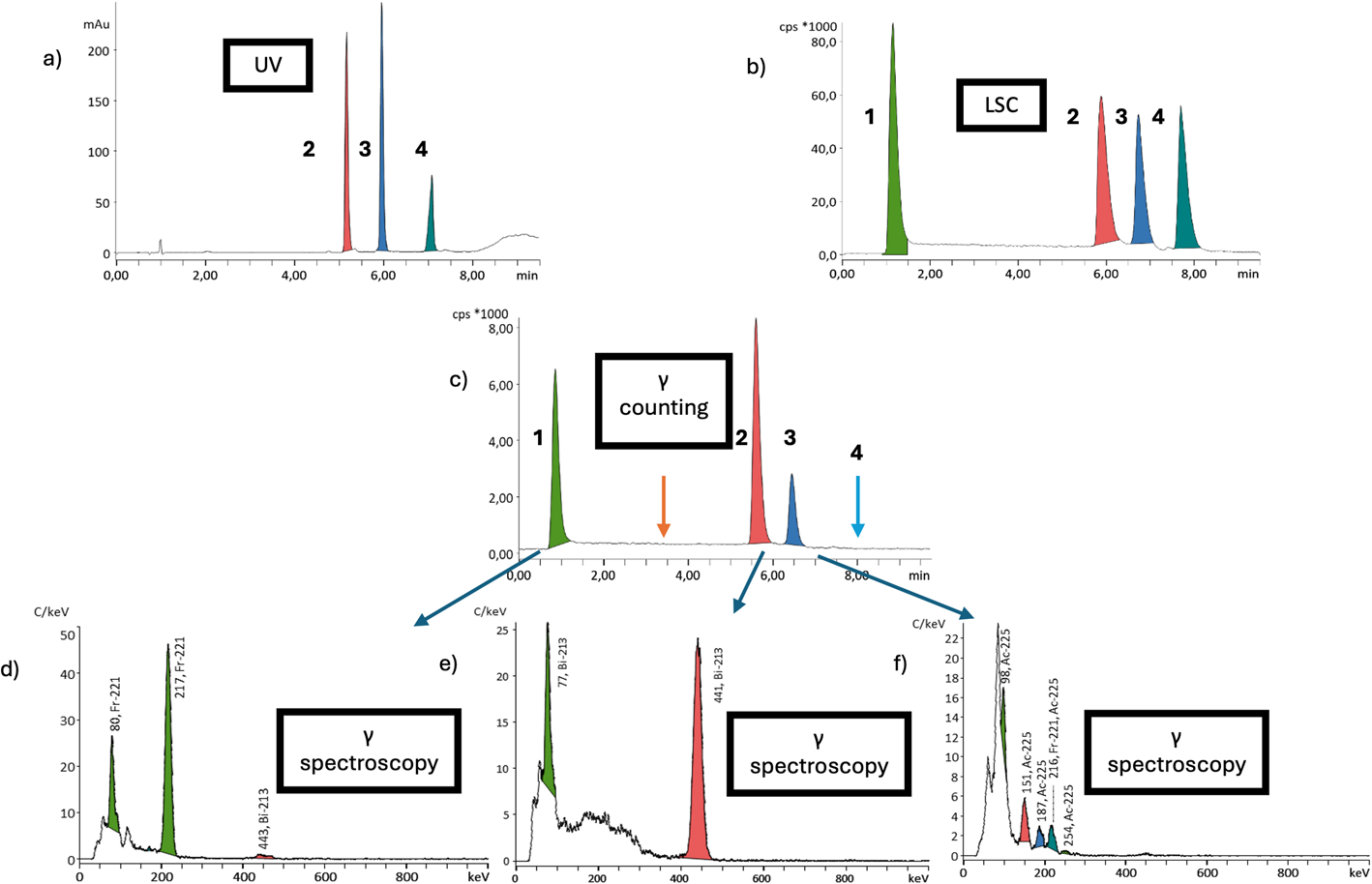
UV-HPLC chromatogram of non-radioactive standards **2** (Bi-PSMA I&T), **3** (La-PSMA I&T as non-radioactive Actinium surrogate) and **4** (Pb-PSMA I&T) (chromatogram a). Using liquid scintillation (chromatogram b), all alpha and beta emitters of a ^225^Ac-decay chain could be observed—an elevated baseline (highlighted by an orange arrow) was observed prior to elution of the [^225^Ac]Ac-PSMA I&T complex (**3**). This elevated baseline is attributed to [^225^Ac]Ac-PSMA I&T partially decaying on the column during its elution and recoiling to ^221^Fr, with the signal returning to its initial level once the complex has eluted. The assignment of **4** was done by comparison of the LSC and gamma-trace (chromatogram c), the peak being absent in the latter chromatogram thus attributed to the pure beta-emitter ^209^Pb (highlighted by the blue arrow). Using inline gamma-spectroscopy **1** could be attributed predominantly to free ^221^Fr with at most a minor contribution of free ^213^Bi (spectrum d), **2** to [^213^Bi]Bi-PSMA I&T (spectrum e) and **3** to [^225^Ac]Ac-PSMA I&T (spectrum f), matching the UV based assignments

Freshly synthesized [^225^Ac]Ac-PSMA I&T was measured using the dual detection setup combining liquid scintillation detection and inline gamma-spectroscopy (Fig. 2). The ^225^Ac used in this study had been separated from ^229^Th on the previous day and therefore already contained daughter radionuclides at the time of radiolabeling. HPLC measurements were typically performed approximately 4–5 h after completion of radiolabeling. Chromatograms obtained from the gamma- and scintillation channels showed complementary profiles (Fig. 3). Three peaks were observed in the gamma-trace, whereas four peaks were visible in the scintillation trace.

The peak with the longest retention time was attributed to [^209^Pb]Pb-PSMA I&T, as it was detected only in the scintillation trace and was absent in the gamma-chromatogram. The assignment was confirmed by fraction collection followed by liquid scintillation counting. Post-fractionation LSC was only performed on the ^209^Pb-containing fraction. The recorded spectrum showed the characteristic beta-emission of ^209^Pb with the expected Eβ_max_ (see SI Fig. S3) (Suliman et al. 2013).

The gamma-emitting isotopes were identified directly using inline gamma-spectrometric measurement. Peak 1 contained predominantly uncomplexed 221Fr together with at most a minor contribution of free ^213^Bi. Peak 2 was assigned to [^213^Bi]Bi-PSMA I&T and peak 3 to [^225^Ac]Ac-PSMA I&T based on their characteristic gamma-spectra recorded inline. Because isotope-specific detector response factors have not yet been established, no rigorous absolute quantification of the minor ^213^Bi fraction in peak 1 is reported.

Baseline separation between ^213^Bi and ^225^Ac enabled acquisition of a daughter-free gamma-spectrum of ^225^Ac showing its characteristic but weak gamma-lines (Asano et al. 1974). An elevated baseline preceding the elution of [^225^Ac]Ac-PSMA I&T was observed in both detectors and returned to its initial level after the complex had eluted.

To confirm the peak assignments, non-radioactive standards of La-PSMA I&T (as an Ac surrogate), Bi-PSMA I&T and Pb-PSMA I&T were analyzed by UV detection (Boros et al. 2025). The retention times corresponded to those observed in the radioactive chromatograms. Small delays between UV and radio peaks resulted from the dead volume between detectors and were consistent for each compound. The UV trace shown in Fig. 3 corresponds to these non-radioactive reference compounds used for retention-time assignment, whereas radionuclide assignment in the radioactive sample was based on the combination of inline gamma-spectroscopy, liquid scintillation detection, and confirmatory fraction analysis.

**Fig. 4:**
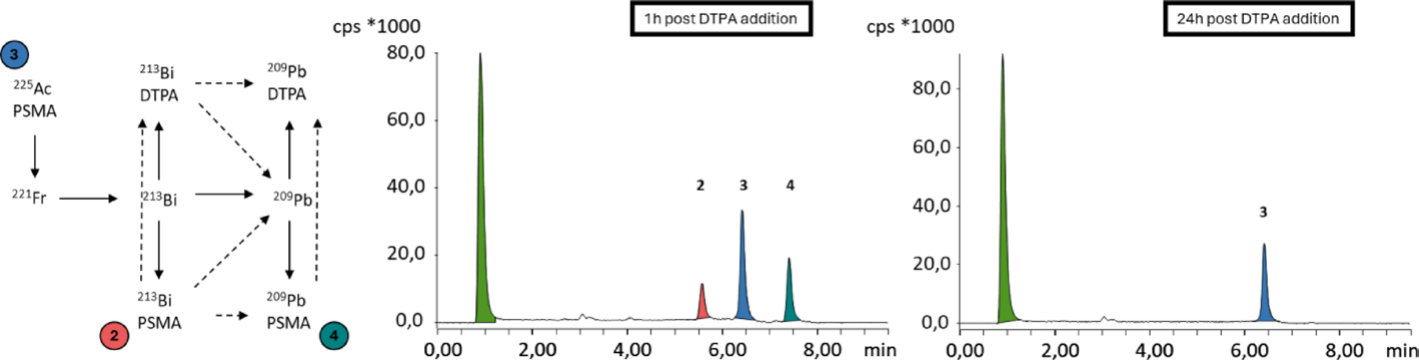
A simplified scheme of the speciation in solution observed within this study. The dashed lines are used where the exact pathway is still unclear. LSC chromatograms of [^225^Ac]Ac-PSMA 1 h and 24 h after the addition of DTPA. NB: The disappearance of [^213^Bi]Bi-PSMA I&T as well as [^209^Pb]Pb-PSMA I&T was only observed once DTPA was added

Addition of DTPA to the reaction mixture altered the observed daughter distribution (see Fig. 4). The [^225^Ac]Ac-PSMA I&T signal remained present, whereas the peaks corresponding to [^213^Bi]Bi-PSMA I&T and [^209^Pb]Pb-PSMA I&T decreased. The amount of ^213^Bi increased in the peak coming at the void volume (see Fig. S4). In contrast to the DTPA-containing sample, the chromatographic profile of a DTPA free reaction remained mostly unchanged over time (see Fig. S5).

## Discussion

The combination of liquid scintillation detection and LaBr_3_-based gamma-spectroscopy enables direct observation of radionuclide distribution within the ^225^Ac decay chain during HPLC analysis. In contrast to conventional approaches relying on fraction collection and delayed gamma-counting after secular equilibrium, this configuration provides real-time information on the chromatographic behavior of both parent and daughter radionuclides. The proposed inline HPLC setup does not require fraction collection for routine speciation. In the present study, fraction collection was used only as an auxiliary validation step to support peak assignment and detector interpretation (see SI Figs. S8 and S9).

The absence of a detectable [^221^Fr]Fr-PSMA I&T complex suggests that the daughter complexes [^213^Bi]Bi-PSMA I&T and [^209^Pb]Pb-PSMA I&T arise from recomplexation processes occurring after recoil release of daughter radionuclides. Francium possesses alkali-metal character with a large ionic radius, and its coordination chemistry remains largely unexplored due to the absence of stable isotopes (Delmau et al. 2013). To our knowledge, coordination of francium by DOTA-type chelators has not been reported. Consequently, recoil-released francium is expected to remain uncomplexed in solution, whereas downstream daughters such as bismuth and lead can recomplex with ligands present in excess.

Recomplexation of daughter radionuclides is facilitated by the large excess of ligand present in the reaction mixture (metal-to-ligand ratio approximately 1:1500 depending on the starting activity). Independent experiments demonstrated that both ^213^Bi and ^209^Pb from a ^225^Ac solution in secular equilibrium form complexes with the DOTAGA-based PSMA ligand at room temperature (see SI Fig. S6), which is consistent with literature reports (Mirzadeh et al. 1993; Maguire et al. 2014; Thiele and Wilson 2018; McNeil et al. 2021). The daughter nuclides initially present at the time of synthesis may contribute to the early composition, but the chromatographic pattern at the time of analysis is also shaped by continued daughter in-growth from the ^225^Ac parent, recoil release, and subsequent recomplexation in solution (see Fig. S5 for a chromatogram measured 24 h after end of synthesis i.e. well after the decay of any initially present daughter isotopes).

The elevated baseline observed prior to elution of [^225^Ac]Ac-PSMA I&T most likely arises predominantly from recoiled ^221^Fr generated continuously from the [^225^Ac]Ac-PSMA I&T complex during the chromatographic run and eluting over a broader time window than the parent complex itself. This interpretation is supported by the inline gamma-spectroscopic data, in which this region showed essentially only the signal attributable to ^221^Fr, and by the observation that the baseline returns to a lower level after elution of the Ac-containing species. The effect was observed in both the gamma and liquid scintillation channels, although the absolute signal intensity differs because of the different detector responses.

Addition of DTPA significantly altered the observed daughter distribution. DTPA is commonly included in radioligand therapy formulations such as [^177^Lu]Lu-PSMA-617 (Pluvicto®) to capture free radiometals and reduce off-target distribution (Hennrich and Eder 2022). In the present experiments the presence of DTPA reduced the formation of [^213^Bi]Bi-PSMA I&T and [^209^Pb]Pb-PSMA I&T complexes, consistent with rapid complexation of recoil-released daughter radionuclides by DTPA. DTPA recomplexed the recoiled daughters which then did not form a new DOTAGA complex. Partial transchelation from the DOTAGA chelator to DTPA may also occur, as previously reported for bismuth complexes (Šimeček et al. 2018). Owing to their high hydrophilicity, these DTPA complexes are not retained on our reversed-phase column and co-elute with the uncomplexed nuclides near the void volume e.g. the formation of a [^213^Bi]Bi-DTPA complex leads to an increase of the signal of ^213^Bi in the first peak (see Fig. S4 for the gamma spectra).

A quantification of the amount of “free” ^213^Bi relative to the amount of [^213^Bi]Bi-PSMA I&T is possible by using a narrow energy range (430–450 keV) centered around the gamma line of ^213^Bi which does not overlap with any nuclide of the decay chain (see Fig. S7). However, a comparison of sensitivity, limit of quantification, and detector-specific response factors for each radionuclide was not established systematically in the present study and will require further studies especially due to the contribution of recoiled ^221^Fr to adjacent signal regions. A comparison of the same samples with the inline LaBr_3_ gamma-detector and the LSC detector showed substantially higher count rates in the LSC channel, consistent with very high response factors known for high energy alpha and beta emitters with LSC. The developed HPLC method therefore provides a useful analytical tool for investigating the redistribution and recomplexation behavior of radionuclides within the ^225^Ac decay chain. Such information may be relevant for improving analytical protocols and quality control strategies for other alpha-emitting radiopharmaceuticals. At the same time, the present work should be regarded as a proof-of-principle and not yet as a formally validated QC method. Formal quantification and validation will require dedicated studies of specificity, precision, robustness, and sensitivity (Gillings et al. 2020, 2021).

## Conclusion

We could demonstrate that a HPLC system combining LaBr_3_-based gamma-spectroscopy with liquid-scintillation detection enables the resolution of the principal radionuclides of the ^225^Ac decay chain present in our formulation of PSMA I&T. The method distinguishes parent and daughter species and directly reveals changes of the speciation in solution arising from heating or DTPA addition. Work is in progress to apply the same configuration to additional ligands and radionuclides to evaluate the general applicability of this dual-detection approach. This work might contribute towards more informative analytical control of alpha-emitting radiopharmaceuticals.

## Supplementary Information

Below is the link to the electronic supplementary material.

Supplementary Material 1

## Data Availability

The datasets generated during and/or analyzed during the current study are available from the corresponding author on reasonable request.
