# Supplementary material for: Inline gamma spectroscopy and liquid scintillation HPLC reveal daughters in 225Ac-radiopharmaceutical
Source: EJNMMI Radiopharm Chem. 2026 Jul 21;11:49. doi: 10.1186/s41181-026-00476-6 (PMC13391994; doi:10.1186/s41181-026-00476-6)
Supplement: Supplementary file 1 — Supplementary Material 1 [file 41181_2026_476_MOESM1_ESM.docx]

Supporting informations: Inline Gamma Spectroscopy and Liquid Scintillation HPLC Reveal Daughters in ^225^Ac-Radiopharmaceutical

Guilhem Claude*, Matthias Balzer, Winfried Brenner, Frank Bruchertseifer, Alfred Morgenstern, David Thonon, Sarah Spreckelmeyer.

|  | |
| --- | --- |
| **Figure S1**: HPLC UV chromatogram measured at 200nm of a reaction mixture. | |
| 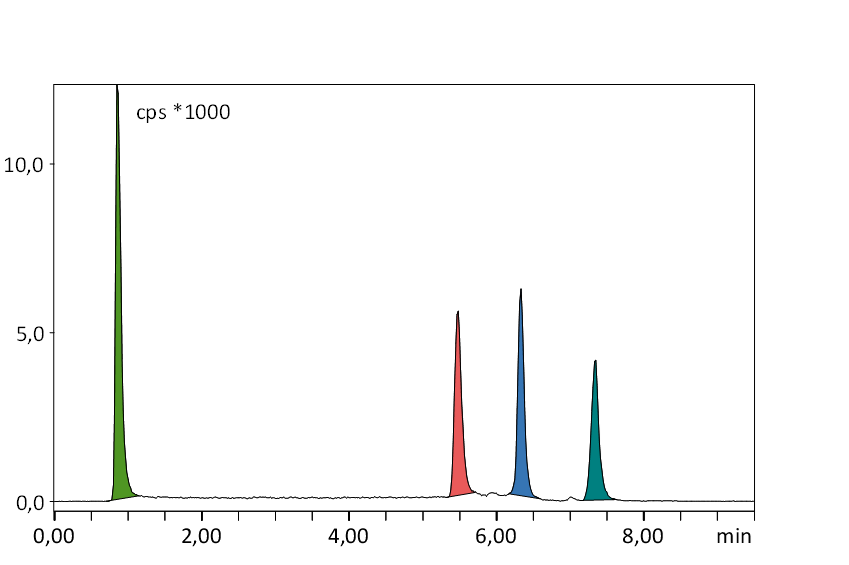 |  |
| **Figure S2:** HPLC chromatogram with LSC detection of a replicate run performed 1h after EOS. |  |

| 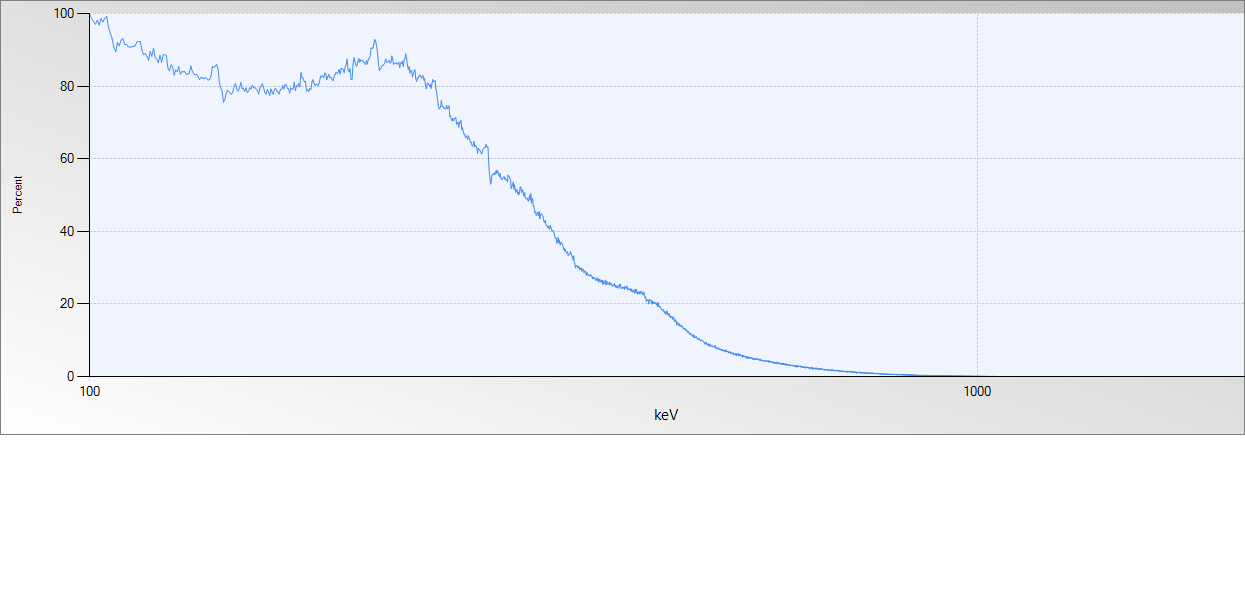 |
| --- |
| **Figure S3:** Liquid scintillation beta spectrum of isolated [^209^Pb]Pb-PSMA-I&T. |


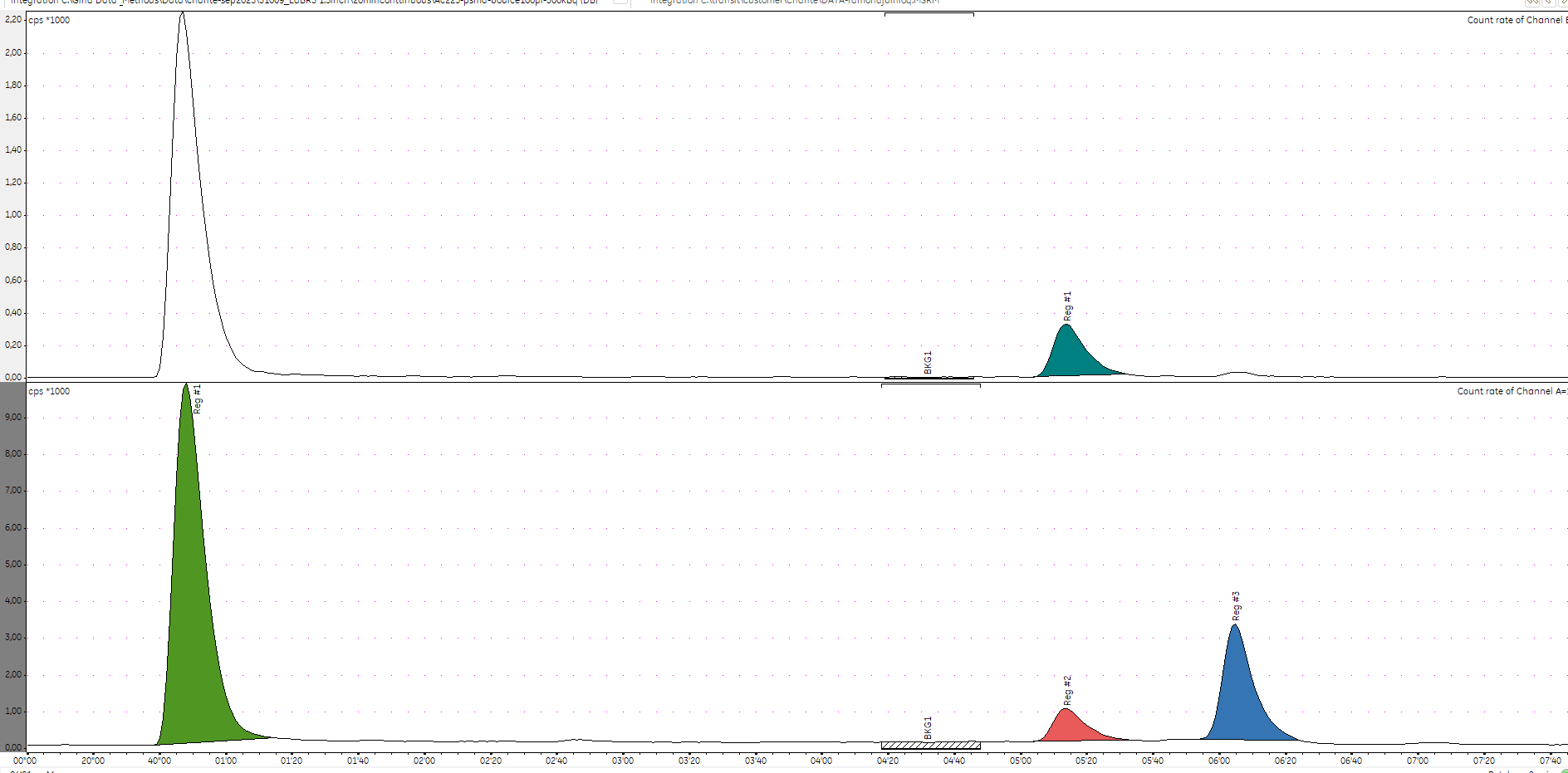


3

2

1

|  |
| --- |
| **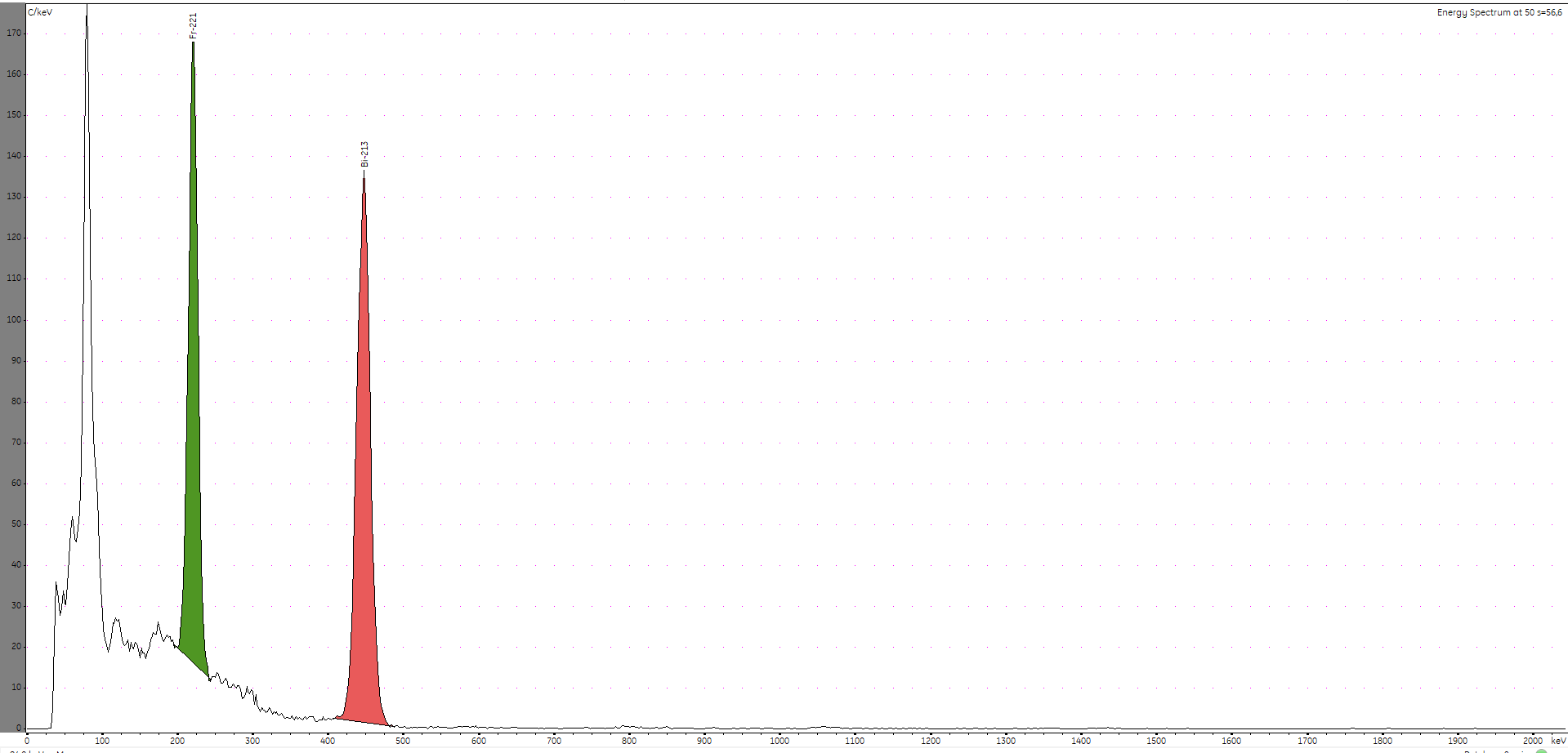**  1  **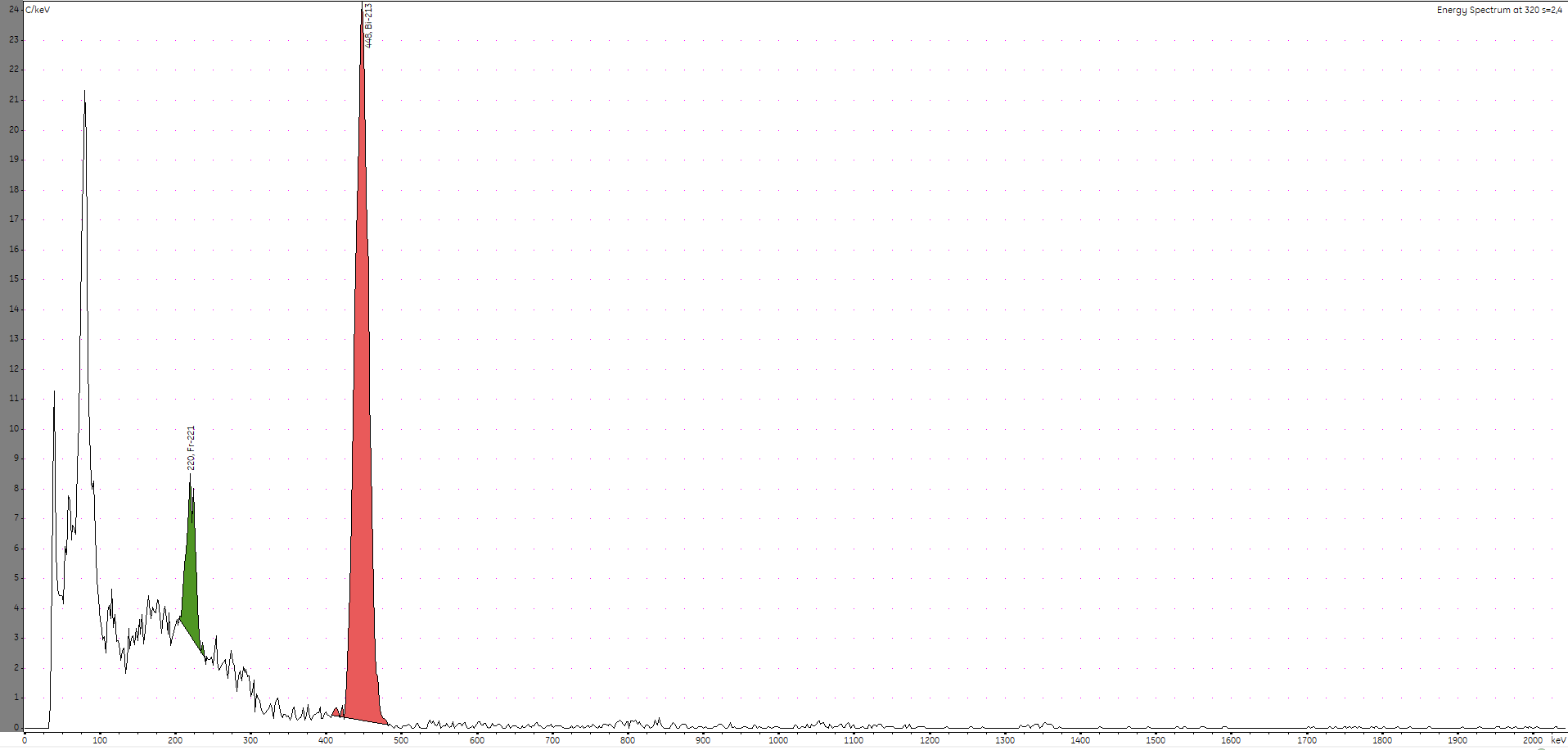**  2  **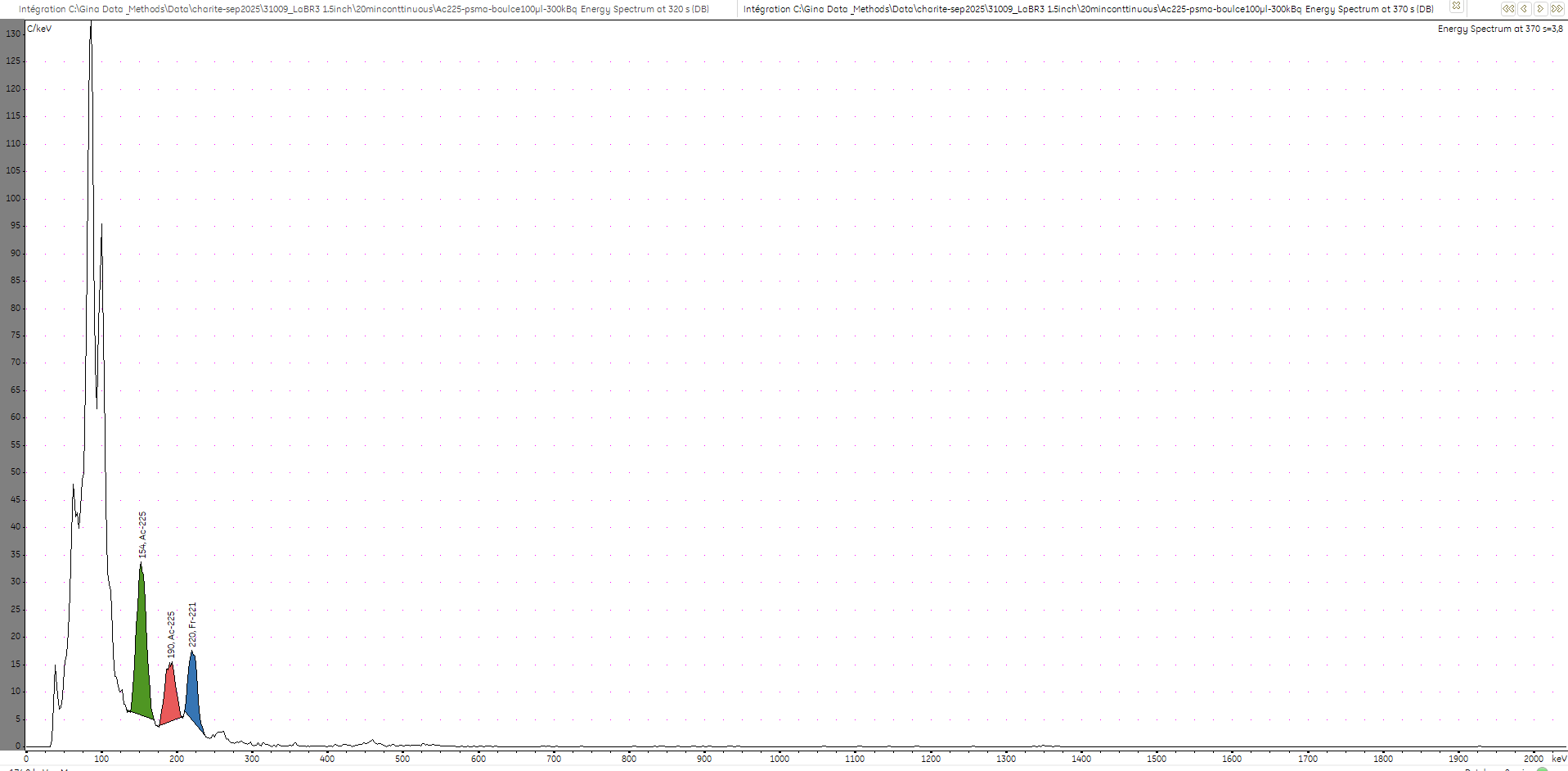**  3  **Figure S4:** HPLC chromatogram with BGO cell (gamma measurement) and gamma spectrum measured inline of every peak after addition of DTPA. A significant amount of ^213^Bi can be detected in the first coming with the void volume which could be caused by a very hydrophilic ^213^Bi-DTPA complex. In the second peak, the ratio of ^221^Fr and ^213^Bi is significantly altered compared to a sample without DTPA as the ^221^Fr caused by the recoiled ^221^Fr coming from the ^225^Ac-PSMA is not diminished while the intensity of ^213^Bi-PSMA peak is now lower. The respective inline gamma spectrum of each peak is depicted below the chromatogram. |

| 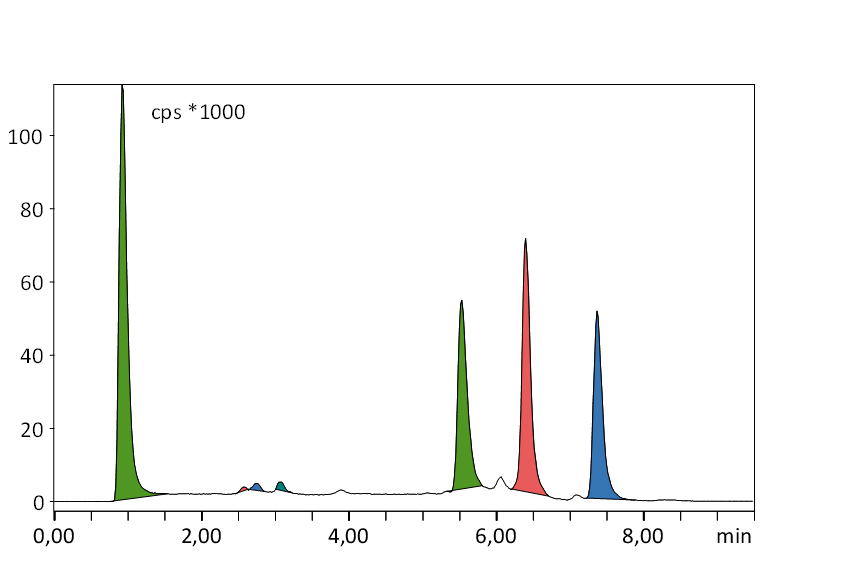 |
| --- |
| **Figure S5:** LC chromatogram with LSC detection performed 24h after EOS. |

|  | |
| --- | --- |
| **Figure S6:** HPLC chromatogram of reaction mixture prior and after heating. | |
|  |  |
|  |  |

| 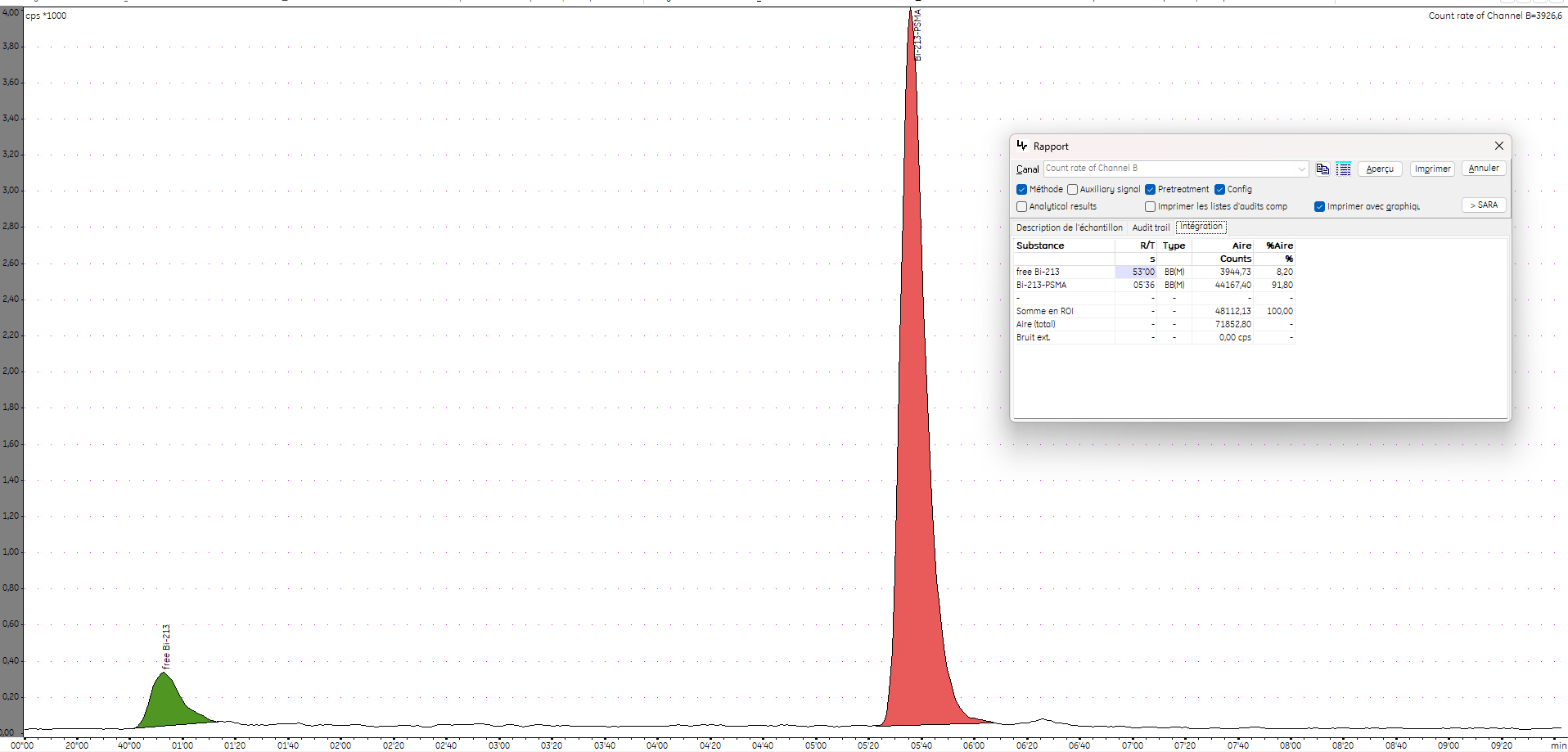 |
| --- |
| **Figure S7**: HPLC chromatogram with high energy window 430-450keV which only shows ^213^Bi containing species. The relative amount of free ^213^Bi could thus be estimated. |

|  |
| --- |
| **Figure S8**: Gamma spectrum of [^213^Bi]Bi-PSMA-I&T measured after fraction collection. A strong ^213^Bi signal is observed, while the ^221^Fr is much weaker. |

|  |
| --- |
| **Figure S9**: Gamma spectrum of [^225^Ac]Ac-PSMA-I&T measured after fraction collection. The pattern of ^221^Fr and ^213^Bi is characteristic of a solution of ^225^Ac in secular equilibrium. |
